# Special Issue on the COVID-19 Pandemic and Psycholinguistic Research: A Call for Papers

**DOI:** 10.1007/s10936-020-09704-9

**Published:** 2020-05-21

**Authors:** Rafael Art. Javier, Marko Lamela, Aubrey Faber, Yosef Amrami

**Affiliations:** grid.264091.8 0000 0001 1954 7928 St. John’s University, New York, USA

## Challenges of COVID-19 Factors to the Academy

Diseases related to Coronavirus-19 and their consequences (APS 2020; Hawryluck et al. 2004; Horesh and Brown 2020; WHO 2020a, b) are forcing a realignment of how we perceive and assess our perception, how we understand and respond to our environment, and how we engage with one another. A resulting challenge for our academy has to do with how we engage remotely in our academic/scientific endeavors likely to impact on research methodology that we now need to reconsider related to subject/sample selections, types of instruments to test our hypothesis, data collection and their interpretation, IRB consideration, etc. In the field of psycholinguistic research such a condition may complicate matters in studies involved in a careful and detailed examination of language processing and production in a virtual environment that is not always reliable and predictable. For instance, we may not be able to clearly observe nonverbal information, and the instruments used to measure our experimental conditions may malfunction or be thrown off line at a critical moment in our data gathering. Moreover, data gathered at this point and subsequently may be affected by emotional/trauma related to immediate and long term consequences of COVID-19 factors, as suggested by previous findings (LoBlue et al. 2019; Morales et al. 2017; Woodruff-Bourden et al. 2002; Campbell 2002; CDC 2014; Murray et al. 2016; WHO 2013).

These conditions may also provide us with a unique opportunity to engage in studies that may be able to provide important and crucial information to understand the various ways and the extent to which the COVID-19 and its consequences may be impacting on language function and cognition, development of linguistic categories, cross-language learning, language-specific concept formation, memory and retrieval of specific events, the role of emotions on language production, etc.

### A Call for Proposals

In response to this challenge, we are launching a call for specialized contributions meant to help address some of these challenges. We are accepting contributions in a form of special issues or individual manuscripts focusing on research and theoretical constructs geared at advancing and guiding research methodology for a variety of topics related to psycholinguistics. Below are some of the themes to consider:

- Semantic analysis of medical misinformation on social media during the Covid-19 pandemic
- Using public figures as case studies for how language has changed during the course of the pandemic. Using their social media posts over time to provide linguistic data.
- Analysis of pandemic narratives. Asking people to speak about their experience of being isolated, how their memory (for what category) may be affected, what gives them comfort, changes in perceptions and language expression, etc.
- Linguistic content analysis of individuals with or without a previous history of trauma
- Issues related to differential effect of increased emotional concern (fear/anxiety, etc.) on language learning and language performance in children, young adults, and the elder populations
- Impact of change in attentional preoccupation on language functioning
- Impact of COVID-19 on specific mechanism involved in reading, writing, concept formation, abstract thinking, etc.
- Difference and quality of metaphor and changes in dimension of metaphor in general population, children, elderly in relation to the Coronavirus experience
- Metaphor interpretation and reading ability
- Organization in speech events
- Speech perception in its multilevel processing system
- Coding and sentence retrieval
- Changing in propositional reasoning with or without proposition
- Development of young children’s awareness of words, syntax, grammatical structures, etc.
- Children’s conversational repairs
- Parsing strategies and discourse context
- Changes in comprehension of complex sentences: A Developmental analysis
- Function of stages in the acquisition of syntax
- Impact of emotional conflicts in voluntary and involuntary component of speech
- Processing of idioms with multiple meanings
- Effect of gender and topic on speech processing, production, styles
- Semantic and syntactic processing in monolingual and bilinguals
- Conditional reasoning and semantic context
- Understanding and processing linguistic time sequence
- Children’s Knowledge of propositional phrase structure
- Children’s concept of word
- Identifying deception in language communication
- Changes in electrophysiological correlates of semantic features
- Impact of increased emotionality and ambiguity in Dyslexic individuals’ reading and language processing/production
- Impact of ambiguity on language perception and judgment of intentionality in normal and paranoid subjects
- Changes in linguistic markers: Concepts of health, illness, and disease
- Semantic and pragmatic factors in the representation of near and far or spatial dimensions
- Language acquisition and children’s representation of knowledge
- Perception of stress contrasts in semantic and nonsemantic contexts by children and adult
- Changing in mentalism and language production
- Bilingual frequency encoding
- Input/output switch in bilingual code-switching of ordinary and emotionally-laden content
- Effect of stress on linguistic generalization in bilinguals
- Language processing and language organization in bilingual population
- Speed perception: Word recognition in foreign language
- Processing of COVID-19 experience among the deaf and mute populations
- Semantic and phonemic verbal fluency in blinds
- Etc.

For any questions about the special issue or suitability of a manuscript, please feel free to contact the Editor-in-Chief at javierr@stjohns.edu or initiatives@stjohns.edu

### Proposal Submission Process

We are requesting a brief abstract of no more than 500 words clearly stating the specific relevance of the manuscript in addressing the contribution of psycholinguistic science in advancing our understanding of the impact of COVID-19 on language processing and production, neurolinguistic issues likely to be affected, impact on psycholinguistic research, etc. We welcome innovative linguistic models likely to encourage new ways of fulfilling our scientific responsibility in virtual contexts while protecting the scientific integrity of the academy. Those submitting a special issue topic should include a brief abstract for each of the contributions to be included in that special issue with the information listed below.

Please, include a cover letter providing names, email addresses and other relevant contact information for three independent reviewers (i.e., individuals who are not connected to the authors in any way). Reviewers from different career stages, institutions, and countries are encouraged. It should also include the following to be submitted to the Editor-in-Chief, Rafael Art. Javier (javierr@stjohns.edu and initiatives@stjohns.edu).

## Important Deadlines

- Abstract submission: June 1, 2020.
- Submission of complete manuscript: October 1, 2020.

When submitting your proposal (s), include the following for each manuscript, in addition to the cover letter:

1. Title of the manuscript (s)
2. List of author (s) and affiliation (s)
3. Abstract (s)
4. Estimated time of completion

Accepted manuscripts will be electronically published on an ongoing basis. The first special issue is expected to be published by June 2021. Subsequent special issue publications will follow, if appropriate.
